# Developing a Global One Health Workforce: The “Rx One Health Summer Institute” Approach

**DOI:** 10.1007/s10393-020-01481-0

**Published:** 2020-07-19

**Authors:** Amanda M. Berrian, Michael Wilkes, Kirsten Gilardi, Woutrina Smith, Patricia A. Conrad, Paulina Zielinska Crook, James Cullor, Thierry Nyatanyi, Martin H. Smith, Rudovick Kazwala, Jonna A. K. Mazet

**Affiliations:** 1 One Health Institute, School of Veterinary Medicine, University of California, 1089 Veterinary Medicine Drive, Davis, CA 95616 USA; 2 Department of Veterinary Preventive Medicine, College of Veterinary Medicine, The Ohio State University, Columbus, OH USA; 3 School of Medicine, University of California, Davis, CA USA; 4 Sokoine University of Agriculture, Morogoro, Tanzania; 5 Global Programs, School of Veterinary Medicine, University of California, Davis, CA USA; 6 Department of Population Health and Reproduction, School of Veterinary Medicine, University of California, Davis, CA USA; 7 Department of Global Health and Social Medicine, Harvard Medical School, Boston, MA USA

**Keywords:** One Health, Experiential learning, Interprofessional education, Professional development, Competency-based curriculum, Program evaluation

## Abstract

The One Health approach has gained support across a range of disciplines; however, training opportunities for professionals seeking to operationalize the interdisciplinary approach are limited. Academic institutions, through the development of high-quality, experiential training programs that focus on the application of professional competencies, can increase accessibility to One Health education. The *Rx One Health Summer Institute*, jointly led by US and East African partners, provides a model for such a program. In 2017, 21 participants representing five countries completed the *Rx One Health* program in East Africa. Participants worked collaboratively with communities neighboring wildlife areas to better understand issues impacting human and animal health and welfare, livelihoods, and conservation. One Health topics were explored through community engagement and role-playing exercises, field-based health surveillance activities, laboratories, and discussions with local experts. Educational assessments reflected improvements in participants’ ability to apply the One Health approach to health and disease problem solving, as well as anticipate cross-sectoral challenges to its implementation. The experiential learning method, specifically the opportunity to engage with local communities, proved to be impactful on participants’ cultural awareness. The *Rx One Health Summer Institute* training model may provide an effective and implementable strategy by which to contribute to the development of a global One Health workforce.

## Introduction

Over the next decade, the United Nations Sustainable Development Goals (SDGs) that encompass social development (e.g., poverty), the environment (e.g., climate change), and economic progress (e.g., infrastructure) are positioned to set the agenda for global health; these goals embody a One Health (OH) strategy (Gostin and Friedman 2015). Implementing an OH approach to problem solving at the interface of people, animals, plants, and ecosystems increasingly demands knowledge, skills, and expertise from diverse disciplines and requires discipline-based experts to work collaboratively (Nyatanyi et al. 2017).

Universities are uniquely positioned to translate OH from concept into action through collaborative training; however, disciplinary inertia, crowded curricula, time constraints that vary by professional training program, and lack of funding for curriculum development and delivery often serve as barriers to progress (Barrett et al. 2011). Furthermore, evidence suggests that training programs that focus on application of concepts, rather than simply theoretical knowledge, are more successful for professional development (Vink et al. 2013).

For veterinary students, a number of experiential short courses have been developed to expand their knowledge, skills, and mentors in OH topic areas, such as AQUAVET^®^, MARVET, CONSERVET, and the Envirovet Summer Institute which ran from 1991 to 2010 (Conrad et al. 2009; Gilardi et al. 2004; Schwind et al. 2016). In medical schools, international clinical rotations have been associated with a deeper appreciation for global public health issues and cross-cultural competencies; yet still relatively few (24%) US medical students participate in global health experiences, and this number has been on the decline (Drain et al. 2007; AAMC 2019). There remains a scarcity of meaningful international opportunities and even fewer comprehensive OH training opportunities (Davis et al. 2017; Mazet et al. 2006; Reid et al. 2016; Rwego et al. 2016; Sikkema and Koopmans 2016; Wu et al. 2016). Few training programs in the USA or around the world offer capacity strengthening programs that are truly interdisciplinary, especially in “hotspot” regions for emerging and re-emerging infectious diseases like East and Central Africa (Amuguni et al. 2017).

High-quality, experiential training programs that focus on the application of professional competencies and provide a forum for dynamic, collaborative problem solving with diverse participants can build key skills for global health practitioners. The *Rx One Health Summer Institute* provides a model for such a program. The main goal of *Rx One Health* is to provide a “prescription” for addressing OH problems, enabling participants to enter global health careers with a more integrated, collaborative perspective. The program is coordinated by the University of California, Davis, in the USA and Sokoine University of Agriculture in Tanzania and involves other partners in East Africa. The curriculum emphasizes the core OH competency domains (Frankson et al. 2016): transdisciplinary collaboration, leadership, discovery, innovation, problem solving, communication, professionalism, diplomacy, teamwork, systems thinking, and cultural capacity strengthening (not only addressing the culture of local communities but recognizing the cultural components of one’s own professions).

Herein, we provide an overview of the *Rx One Health* program approach and development (i.e., steps taken to create a professionally relevant experiential program in OH for adult learners) and describe the inaugural course (2017) with an evaluation of the impact of the course on participants’ OH competencies. Educational assessments were conducted as part of the curriculum and are reported here for quality improvement and program evaluation purposes.

## Methods

### Curriculum Design and Development

*Rx One Health Summer Institute* was grounded in the pedagogical framework of a competency-based curriculum. Schools of medicine, public health, and veterinary medicine have been actively moving to competency-based educational models, and this paradigm is particularly relevant for collaborative problem solving and critical reasoning (Frenk et al. 2010). Overall *Rx One Health* program goals included enabling participants to:

1. Describe how an OH approach can be operationalized to respond to complex health problems;
2. Discuss OH problems from a transdisciplinary perspective;
3. Describe methods to implement and evaluate OH approaches to address common problems;
4. Facilitate effective communication, collaboration, and collegiality among participants with different professional and cultural backgrounds;
5. Strengthen professional networks across health disciplines;
6. Design, monitor, and evaluate OH research and community engagement; and
7. Identify social, ecological, and economic barriers that might impact an OH approach.

Aligned with these goals, instructors developed learning experiences using the backward design method (Wiggins and McTighe 2005). This three-step process involved identifying desired competencies, determining acceptable evidence of learning, and planning learning experiences and instruction. Competencies were developed within five curriculum thematic areas which encompassed technical (e.g., outbreak investigation) and cross-cutting (e.g., communication, leadership) OH domains: (1) OH Foundations; (2) Zoonotic Disease; (3) Wildlife Health and Stakeholder Engagement; (4) Research Methods and Education; and (5) OH Policy, Systems, and Solutions (Table 1). Given the experiential nature of the curriculum, authentic assessment (i.e., demonstration of real-world tasks) was primarily used to provide evidence of learning. As such, learning experiences were designed to provide participants with opportunities to practice and demonstrate skills.

**Table 1:** *Rx One Health Summer Institute* Curricular Themes, Learning Objectives, and Examples of Planned Learning Experiences.

Curriculum thematic areas	Learning objectives	Examples of learning experience (location)
Theme 1: One Health foundations	Define One Health as an organizing principle and list advantages of a One Health approach to problem solving compared to traditional siloed approaches	Discussion: An introduction to One Health as an organizing principle, perspective, and approach (T)
	Identify core competencies for One Health practitioners compared to traditional content experts	Activity: Developing a professional standard for one health practitioners (T)
	Describe advantages and challenges to implementing a One Health approach to problem solving considering local context	Case study: Health for Animals and Livelihood Improvement (HALI) Project (Mazet et al. 2009) (T)
	Draw a diagram to demonstrate the content areas at the intersection of One Health providers	Discussion: Collaboration, networking, and creativity—what really powers global health innovation (T)
Theme 2: Zoonotic disease	Describe the role of the physical environment (landscape, water availability, climate change, etc.) on pathogen transmission at the human–animal–environment interface	Discussion: Global infectious disease and environmental policy from an African perspective (T)
	Describe the role of social and cultural beliefs and traditions on pathogen transmission at the human–animal–environment interface	Stakeholder engagement: Maasai household (T)
	Identify risks to food safety and security caused by emerging and re-emerging infectious disease.	Field exercise: Village Poultry Biosecurity (diagnostic testing, vaccination) and Tour of Veterinary Investigation Centre (T)
	Outline an approach to One Health surveillance of diseases and list barriers to its implementation	Discussion: Control and surveillance for zoonotic diseases and diseases of economic importance in livestock (T)
	Describe ways in which social determinants of health and well-being (e.g., poverty, war, drought) can impact One Health problems	Tour/stakeholder engagement: Mtera fishing community (T)
Theme 3: Wildlife health and stakeholder engagement	For a given One Health problem, identify key stakeholders at the local, national, regional, and global levels and attempt to anticipate their concerns	Discussion: Value chain and stakeholder analysis for smallholder agricultural producers (T)
	Describe the role of rural/indigenous peoples in the management of wildlife and environmental health	Stakeholder engagement: Ruaha Carnivore Project, wildlife connection (T) and gorilla doctors (R)
	Compare One Health implications in varied ecosystems (e.g., terrestrial vs. marine ecosystems)	Case study: Current health concerns in Ruaha National Park (T)
	Describe a process to inform and engage stakeholders, including ways to demonstrate cultural sensitivity, professionalism, and open-mindedness	Stakeholder engagement with community health workers and dairy cow owners (R)
	Demonstrate communication skills for effective community engagement	Activity: Communicating perspective with “Zoom” group exercise (T)
Theme 4: Research methods and education	Safely trap and collect biological samples from live wildlife	Field exercise: Wildlife health surveillance (bats, rodents, giraffes) and non-invasive sampling (non-human primates) in Ruaha National Park (T)
	List four research approaches that are used in One Health and describe the advantages and limitations of each	Discussion: Community-based research methods (T)
	Design and conduct a community-based research plan to study a One Health problem	Field exercise: Design a qualitative research plan to identify ways to protect gorillas from local destruction of environment and from ecotourism transmission of human diseases (R)
	Describe the tenants of adult learning and apply them to engage a community around a One Health project	Field exercise: Observe Rwanda’s attempt to reduce transmission of HIV (R)
Theme 5: One Health policy, systems, and solutions	Describe existing government infrastructure that is responsible for monitoring health problems and list the advantages and disadvantages	Tour/Discussion: Ifakara Health Institute and Public Health Research (T)
	Outline the advantages of having government develop policies to manage One Health problems using an integrated, centralized, multi-disciplinary approach	Case Study: Emerging and zoonotic infectious diseases and outbreak management (T)
	Evaluate an existing government policy using evidence to support your perspective using a SWOT approach	Activity: Role of government in promoting dairy consumption by small families (R)
	Develop an iterative learning approach to innovation and provide a specific, practical, acceptable, measurable solution to a One Health problem (e.g., human–wildlife conflict, food quality/safety, zoonotic disease transmission)	Activity: Brainstorm and discuss with experts and community members problems, solutions, and potential barriers to address the domestic animal/wildlife interface while promoting economic growth for local communities (R)

*T* Tanzania.

*R* Rwanda.

*SWOT* Strengths, weaknesses, opportunities, threats.

### Program Leadership and Faculty

*Rx One Health* facilitators and instructors came from Tanzanian and Rwandan academic institutions (Sokoine University of Agriculture, University of Rwanda), governmental (e.g., Tanzania National Parks) and non-governmental organizations (e.g., Ifakara Health Institute, Gorilla Doctors), as well as University of California Davis’ Schools of Veterinary Medicine (SVM) and Medicine (SOM). Instructors represented diverse fields, including agriculture, environmental policy, veterinary medicine, human medicine, and conservation and were selected based on their subject matter expertise and alignment with program goals. Prior to developing their learning experiences, all instructors received a training handbook which outlined course goals, learning theory, tools for effective facilitation, and student biographies to improve the integration of learning objectives and engagement of learners in the community.

### Program Organization and Implementation

#### Pre-immersion Preparation

*Rx One Health* participants independently completed a series of preparatory readings, tutorials, and assessments that enabled instructors to assess their capabilities prior to the in-country sessions (USAID PREDICT 2018). Additionally, participants received a global travel guide that provided country-specific recommendations for cultural sensitivity, health, and safety/security. Materials were available electronically via a secure, cloud-based file-sharing platform. Participants also had the opportunity to participate in synchronous Web-based group video conferencing to discuss travel logistics and ethical considerations for short-term international experiences with instructors as well as to facilitate communication among participants prior to arrival in country.

#### In-Country Sessions

The in-country program was divided into two sessions: Session 1 (Tanzania) and Session 2 (Rwanda). Each session spanned 2 weeks and presented OH challenges, stakeholders, and solutions within the context of the local setting. In each session, the curriculum focused on multiple thematic areas and learning objectives therein (Table 1). Learning objectives and associated competencies addressed disciplinary and technical health knowledge, social/cultural/historical perspectives of complex and emerging health problems, as well as cross-cutting professional characteristics (e.g., communication, ethics, and diplomacy). The program aimed to build these competencies through an increasingly learner-centered curriculum.

Session 1 (June 4–18), held in Tanzania (Dar es Salaam, Iringa, Ruaha National Park, Morogoro, and Bagamoyo), focused on OH foundations (e.g., interest-based problem solving that uses a process of consensus to find solutions that meet the needs of all parties); health monitoring of livestock and free-ranging wildlife; stakeholder engagement to understand health and economic challenges of local communities; and methods in environmental, infectious disease, and community-based research. Learning experiences included classroom-based discussions, hands-on field exercises (e.g., bat and rodent trapping and sampling), tours and demonstrations (e.g., sample processing and diagnostics), and small group problem solving and role playing (e.g., disease outbreak investigation simulation) which continually reinforced communication, organization, collaboration, and conflict resolution skills. Examples of learning experiences and the location of field exercises are provided in Table 1.

Session 2 (June 19–30), held in Rwanda (Kigali and Kinigi), continued to emphasize OH foundations, cultural capacity building, leadership skills, human healthcare delivery, and communication with an emphasis on stakeholder engagement. Additionally, this session introduced the concepts of community health, human resource capacity building, food safety, commercial animal agriculture, and public health (e.g., human causes of morbidity and mortality and opportunities for intervention) in preparation for a capstone immersion exercise. This exercise challenged participants to actively acquire on-site information from local stakeholders on OH challenges (e.g., child nutrition, community health challenges, livestock welfare and production, wildlife conflict, economic deprivations) to develop creative and integrative solutions to address some of these challenges. Participants worked together to develop and deliver an oral presentation to propose their OH solutions to a group of governmental and non-governmental stakeholders who provided feedback and discussed the intricate barriers and existing opportunities for implementing proposed solutions.

### Program Evaluation

#### Qualitative Assessment

At the start of the in-country session, each participant proposed “Professional Responsibilities for OH Practitioners” (pre) to outline their ideas for core competencies for OH professionals. Responsibilities were updated as the course progressed, and at the end of the program, participants submitted their modified ideas (post) for discussion. Pre- and post-program responsibilities were transcribed, and responses were analyzed inductively using the constant comparison method (Dye et al. 2000) to determine how participants’ understanding of OH competencies changed after completing the course. Participant responses which represented key themes were selected for inclusion in the manuscript. A word cloud generator (TagCrowd.com) was also used to determine and compare the frequency of terms.

#### Quantitative Assessment

A pre-/post-self-efficacy assessment, administered at the end of the in-country session, compared participants’ perception of their own growth in understanding and abilities related to *Rx One Health* competencies before (pre) and after (post) the program. Assessments included nine paired questions each with five response categories ranging from “Poor” to “Excellent.” This retrospective format, where before and after information is collected concurrently, was chosen to reduce response-shift bias, a source of contamination of self-report measures that often results in overestimation of pretest scores (Raidl et al. 2004).

Descriptive statistics, including frequency, median, and range, were used to describe participant demographics. Mean, standard deviation, and paired t-tests were used to compare mean scores for each objective (9) on self-efficacy assessments. All quantitative analyses were conducted using SAS v9.4 (SAS Institute, Cary, NC, USA), and a *P* value of ≤ 0.05 was considered significant.

## Results

### Demographics of Participant Cohort

*Rx One Health Summer Institute* 2017 provided training to 21 (10 males, 11 females) participants. Five countries were represented, including the USA (*n* = 11), Rwanda (*n* = 5), Tanzania (*n* = 3), Denmark (*n* = 1), and Nepal (*n* = 1). The median age of participants was 26 (min = 23, max = 54). The participants were early career professionals and students representing the fields of veterinary medicine, biological sciences, natural resources management, public health, human medicine, agricultural development, and community development.

### Qualitative: “Professional Responsibilities for OH Practitioners”

At the start of the training course, participants more frequently emphasized their individual knowledge and skills to solve problems affecting humans, animals, and the environment. At the conclusion, participants more often expressed OH competencies in terms of generating collaboration and communication across multiple disciplines and with communities (Table 2). Before the training, participants described OH challenges from a narrow, disease-centric perspective, particularly zoonotic diseases. At the conclusion, participants described OH challenges more broadly, emphasizing such challenges as human–wildlife conflict, human and animal welfare and well-being, and ecosystem health. When cultivating solutions to these challenges, participants emphasized sustainability, respect, interdisciplinarity, and cultural sensitivity.

**Table 2:** Participant Ideas of Professional Responsibilities for One Health Practitioners Before (Pre) and After (Post) Participating in the *Rx One Health Summer Institute*.

	Pre-training	Post-training
Participant 1	“Use my knowledge and skills to promote the health of humans, animals, and environment”	“Encourage and promote good collaboration and good communication among different disciplines in order to achieve the global well-being of humans, animals, and environment”
Participant 2	“Use my skills for benefit of community”	“No single person, discipline, ministries can be able to address complex health challenges”
Participant 3	“Use my knowledge of One Health to protect humans, animals, and the environment”	“Promote open communication and collaboration between disciplines, particularly in regards to the health and well-being of humans, animals, and the environment”
Participant 4	“Use my knowledge, skills, and understanding to advance the health of communities”	“Discover and learn about the challenges faced by all individuals with a stake in a community, as well as the community’s existing strengths and assets” “Foremost engage and collaborate with the community, never losing sight of the fact that this is their community”

### Quantitative: Pre-/Post-Self-Efficacy Assessment

Participant self-efficacy showed statistically significant improvements across all measured competencies. Those that resulted in the greatest difference between pre-training and post-training were: (1) understanding challenges to the implementation of the OH approach and (2) understanding community-based research and engagement within an OH framework. Complete results are provided in Table 3.

**Table 3:** Retrospective Self-Efficacy Assessment of *Rx One Health Summer Institute* Participants, Using Five-Point Scale (1 = Poor, 2 = Fair, 3 = Neutral, 4 = Good, 5 = Excellent).

*Rx One Health* competency	Pre-training mean (SD)	Post-training mean (SD)	Mean difference (95% CL)	Paired *t*-test (*df*), *P*
Understanding of One Health core competencies	3.16 (0.69)	4.58 (0.51)	1.42 (1.13–1.71)	*t*(18) = 10.21, *P* < 0.0001
Understanding of pathogen transmission dynamics at the human–animal–environment interface	3.26 (0.99)	4.37 (0.60)	1.11 (0.83–1.38)	*t*(18) = 8.49, *P* < 0.0001
Ability to identify and work across stakeholder types	2.53 (0.61)	4.16 (0.69)	1.63 (1.3–1.96)	*t*(18) = 10.4, *P* < 0.0001
Understanding of community-based research and engagement within a One Health framework	2.67 (1.03)	4.56 (0.62)	1.89 (1.48–2.3)	*t*(17) = 9.63, *P* < 0.0001
Ability to create and deliver effective One Health messaging	2.79 (0.79)	4.11 (0.74)	1.32 (1.04–1.6)	*t*(18) = 9.85, *P* < 0.0001
Understanding of wildlife health monitoring methods	2.42 (0.96)	4.05 (0.4)	1.63 (1.2–2.06)	*t*(18) = 7.95, *P* < 0.0001
Ability to apply a One Health approach to health and disease problem solving	3.05 (0.91)	4.53 (0.61)	1.47 (1.18–1.77)	*t*(18) = 10.5, *P* < 0.0001
Understanding of disease surveillance	2.95 (1.03)	4.37 (0.68)	1.42 (0.96–1.88)	t(18) = 6.44, *P* < 0.0001
Understanding of challenges to the implementation of the One Health approach	2.26 (0.87)	4.42 (0.51)	2.16 (1.83–2.49)	t(18) = 13.67, *P* < 0.0001

*SD* standard deviation.

*CL* confidence limit of the mean.

*df* degrees of freedom.

## Discussion

The development and evaluation of the *Rx One Health Summer Institute*, described within, provides an example by which to produce a multi-institutional, interprofessional, experiential training program grounded in the core competencies of OH education. Learning assessments provided support of enhanced participant understanding and ability with regard to research and evaluation methods, particularly at the community level. Assessments also suggested an evolution of thought regarding OH topics, including cultural competence and interdisciplinary collaboration. After the training, participants more readily expressed OH competencies in terms of generating collaboration and communication across multiple disciplines. In essence, participants became more aware of the need to build “essential networks” to create and sustain the human assets necessary to implement an OH community network.

There was also a clear broadening of participants’ definition of OH challenges and mechanisms by which to address them. There was a shift from a disease-centric perspective of OH challenges to one that more readily considered health in terms of the community—human, animal, ecosystem, and economic well-being. Given the origins of the OH concept in the management of shared disease threats to humans and animals (Zinsstag 2012), the emphasis on zoonotic diseases was not unexpected. However, the disciplinary diversity of the *Rx One Health* program’s curriculum, instructors, and participants likely contributed to the broadening of perspectives demonstrated.

Building international collaborations and professional networks is an essential component for addressing global health challenges (Barrett et al. 2011). The multi-institutional, multi-national representation of *Rx One Health* instructors and participants facilitates the development of a network of solution-oriented global health professionals, and with each cohort, the network grows. Future iterations of the *Rx One Health* program may benefit from an even greater representation of disciplines and home countries among participants. In particular, participants from low- and middle-income countries (LMIC), where the likelihood of emerging infectious disease threats are high and human and physical resources are most constrained (Jones et al. 2008; Vink et al. 2013), should be prioritized.

Operationalizing the OH approach requires that current and future health professionals train with a focus on cross-cutting issues, such as zoonotic diseases, public health, environmental degradation/climate change, economics, risk assessment and surveillance, and policy development (Vink et al. 2013). International working groups have identified OH competencies meant to guide educational development (Togami et al. 2018). Education through practical experience, addressing real-world problems, is the basis of OH capacity strengthening and the movement from theory to practice. In North America, there has been a significant shift in defining OH as core educational material in professional health programs. The North American Veterinary Medical Consortium identified OH knowledge as a core competency for veterinarians in 2011 (Amuguni et al. 2017). Similarly, the American Medical Association has endorsed the OH concept (Stroud et al. 2016). Most recently, the Council on Education for Public Health added OH to the accreditation criteria for US public health schools (Togami et al. 2018).

Future iterations of the *Rx One Health Summer Institute* should include the regular review of the curriculum to consider topics underrepresented in these formal OH educational offerings, such as social sciences, plant biology, antimicrobial resistance, and environmental law and policy (Togami et al. 2018). The timing and duration of the program should also be carefully considered to be inclusive of students concurrently enrolled in degree programs. It may be that these sorts of multi-disciplinary field programs would be best directed at final year professional or graduate students or graduates of health science programs, although securing time away from work may be problematic for some participants after completing degree programs and entering the workforce. To increase rigor of future program evaluations, results of multiple iterations could be combined to increase sample size and assessments could be collected from the same cohort longitudinally to help determine how training may translate to improved proficiency in the workplace.

Developing a workforce capable of anticipating and responding to global health challenges is a top priority. Professional trainings like *Rx One Health* are necessary to fill gaps identified in currently available health and medical programs. Long-term success and sustainability of these trainings must be addressed and include institutional and financial support. Many US agencies, such as the Centers for Disease Control and Prevention (CDC), United States Agency for International Development (USAID), United States Department of Agriculture (USDA), and Department of Defense (DoD), as well as international organizations such as World Health Organization (WHO) and the United Nation’s (UN) Environment Programme, stand to gain from a more integrated and collaborative OH workforce (Barrett et al. 2011).

Education is the basis for OH capacity development (Vink et al. 2013). Overall, the *Rx One Health Summer Institute* provides a model for increasing accessibility to OH education. Its emphasis on applied and practical training provides opportunities for participants to develop communication and problem-solving skills across disciplinary and cultural settings. These types of training programs can augment OH curricular offerings, generating a workforce that is capable of integrating OH competencies into public and global health practice at multiple levels and converting them into meaningful action.

## Conclusions

Intensive short courses, such as the *Rx One Health Summer Institute*, provide an effective and implementable strategy by which to contribute to the development of a global workforce capable of collaborating with communities and other stakeholders to solve complex health challenges at the human–animal–plant–ecosystem interface. Training programs can be used as a forum for collaboration among health disciplines, allowing for the development of multi-national and multi-cultural cohorts of each year’s students and encouraging the ongoing development of a solution-oriented community of global health professionals, a necessary component for success of the OH approach.
